# A profile of injuries suffered by female soldiers serving in the Australian Army

**DOI:** 10.1186/s12889-022-13225-6

**Published:** 2022-04-23

**Authors:** Ben Schram, Robin Orr, Rodney Pope

**Affiliations:** 1 Tactical Research Unit – Bond University, Bond Institute of Health and Sport, 2 Promethean Way, Robina, QLD 4229 Australia; 2 School of Community Health, Charles Sturt University, Albury, NSW Australia

## Abstract

**Introduction:**

Female soldiers comprise an important component of any modern army, yet little research has been performed to investigate differences in the profiles of injuries reported by qualified female and male army personnel.

**Aim:**

The aim of this study was to compare injury rates and patterns between female and male soldiers of the Australian Regular Army (ARA).

**Methods:**

Data pertaining to all injuries reported by ARA members over a two-year period were accessed from the SENTINEL database and analysed descriptively. Findings regarding injury patterns were reported by most common location, nature, mechanism, and activity being performed at the time of injury. Injury incidence rates (IR) were calculated based on population size, and injury incidence rate ratios (IRR) comparing female and male injury rates were determined.

**Results:**

A total of 8750 injuries were recorded across the two-year time period (2018–2020) of the study (minor injuries: *n* = 1766 female, *n* = 6870 male; serious injuries: *n* = 19 female, *n* = 95 male). Higher incidence rates of minor injuries were reported for female soldiers (IR = 20.75 injuries/100 soldiers/year) when compared to male soldiers (IR = 13.60 injuries/100 soldiers/year), with an IRR of 1.53 [95% CI = 1.46–1.60]. More serious injuries were reported at a similar rate between female (IR = 0.22/100 soldiers/year) and male soldiers (IR = 0.21/100 soldiers/year), with an IRR of 1.05 [95% CI = 0.65–1.72]. Female soldiers tended to report more ankle injuries than male soldiers who reported more knee injuries. Physical training and combat training were the most common causes of injury for both sexes.

**Discussion:**

There were subtle differences in body locations of minor injuries within female and male soldiers. Both minor and more serious injury profiles were otherwise similar between sexes. Therefore, strategies required to minimise injuries in female soldiers may be similar in many respects to strategies required for male soldiers but require some differences to account for the subtle differences in body locations of injury, and so to ensure effectiveness across all personnel.

## Introduction

Numerous military forces around the world are increasing the numbers of females in their armed forces, with many targeting 25–30% representation by female personnel [1]. The valued role of female soldiers is being further enhanced through direct combat roles being opened for female personnel in armies of many countries [2, 3]. To ensure capability is maintained, through an effective fighting force, strategies to optimise soldier performance, and subsequently minimise injury risk are paramount [4].

The Australian Defence Force (ADF) is comprised of both male and female personnel, with numbers increasing from around 15% female representation in 2015 to approximately 18% in 2019 [5]. The ADF is comprised of Navy, Army, and Air Force services, with female personnel being represented the least in the permanent Army (14.2%) when compared to the Navy (21.4%) and Air Force (23%). Despite investigations reporting injuries in Army basic training [6] and sport [7], explorations of near misses and exposures [8], and comparisons of full time and reservist populations [9], no comprehensive comparisons of injury profiles of female and male soldiers have been conducted to date, outside of load carriage injuries [10].

Many studies to date have shown that female military personnel report injuries at a higher rate than their male counterparts [11–13], however, this finding may be influenced by a variety of factors. Injury rates between male and female personnel are similar when fitness is accounted for [11, 14, 15], and injury rates appear to equalize at latter stages of training [16, 17]. Furthermore, female soldiers are considered to be more likely to report an injury that occurs than male soldiers [18]. As such, with injury rates between the sexes being similar once fitness is accounted for, and a propensity for higher reporting of injuries that occur by female personnel, it is possible that female soldiers actually sustain fewer injuries than male soldiers of similar fitness levels.

Any differences in injury rates or types may be due to anthropometric, biomechanical, and physiological variances between male and female military personnel [1]. Therefore, even if similarities (or differences) between the sexes in injury rates exist, requirements for targeted injury reduction programs may differ, and need to be informed by rigorous profiling of common injuries [6], without uninformed assumptions being made of similarities between the sexes. Injury reduction programs which are general in nature may not be the best fit for all personnel, given actual and potential differences between the sexes. The aim of this study was therefore to determine the injury rates of female and male soldiers of the Australian Army and to profile the most common injury types.

## Methods

A cohort study involving analysis of data collected prospectively to inform Department of Defence work health and safety activities was conducted to determine the differences in injury rates and types between female and male soldiers of the Australian Army. Data pertaining to all injuries suffered by Australian Army members was accessed for the two-year period from July 2018 to June 2020, from the SENTINEL database. This timeframe was during a period of minimal operational deployments for the Australian Army and followed the end of large scale operations in Afghanistan. This self-reporting database is the incident reporting database utilised by the Department of Defence which is designed to capture all workplace health and safety incidents within the organisation. The variables of interest for this study were the: date of injury, part of the body injured, injury type, activity being performed, mechanism of injury, nature of injury, sex of the injured person, and geographical location in which the injury was suffered.

Injury data were included if the reported injury was suffered by a current, full-time, serving soldier of the Australian Army who was injured during the period of interest. Data were excluded if the injury was suffered by a non-human defence member (i.e., canine), the injury was to a reservist (part time personnel), the injury was not suffered while on duty, or the incident was classified as anything other than a minor or serious injury (for example, classified as a near miss, exposure, or dangerous incident). For this investigation, and as per the Australian Department of Defence event and severity definitions, a minor injury (MI) was defined as any minor injury which did not result in a fatality, serious injury (SI), illness, or dangerous incident. A SI required immediate treatment as an in-patient in a hospital, with examples including injuries such as amputation of any part of the body, serious head injury, serious eye injury, serious burns, separation of skin from an underlying tissue (such as de-gloving or scalping), spinal injury, or serious lacerations [19].

Some nature of injury categories that were similar were combined to form broader categories, based on their description. For example, injuries to muscles (a soft tissue) and/or tendons (a soft tissue) were combined into the broader category of ‘soft tissue injuries’. A similar approach was used for fractures, with those which did, and did not require surgery, and those which were categorised as ‘minor’, or ‘unspecified’ fractures, combined into the broader category of ‘fractures’. While this approach may have reduced some sensitivity in the data, it did account for minor data entry errors within broad categories and provide for a clearer overall profile of injuries to be developed, an approach which has been used in previous research [6].

Physical training was used as an umbrella term for all of its components, as was combat training. Sport as a generic term was also used to include all sports in which injuries were suffered. Due to the detailed nature of the exported injury data, injury profiles were reported based on top five categories for each variable, with the remainder of categories reported as ‘collated others’.

Underlying Army population sizes were gathered from the Defence Census and were reported as 25,263 male and 4256 female soldiers for the time period covered by the study [5]. Injury incidence rates were calculated per 100 personnel per year for comparison. To calculate this figure, in each instance the number of injuries was divided by the underlying population size multiplied by two (years) and multiplied by 100 personnel. Injury risk ratios (IRR) and 95% confidence intervals (95% CI) using males as the reference were also calculated by dividing the injury incidence for female soldiers by the incidence for male soldiers to generate the IRR and using the following formula for 95% CI:

$$95\%\mathrm{CI}=\exp\ \left(\ln\ \left[\mathrm{IRR}\right]-1.96\ \mathrm{x}\ \mathrm{SE}\ \left(\ln\ \left[\mathrm{IRR}\right]\right)\right)\ \mathrm{to}\ \exp\ \left(\ln\ \left[\mathrm{IRR}\right]+1.96\ \mathrm{x}\ \mathrm{SE}\ \left(\ln\ \left[\mathrm{IRR}\right]\right)\right)$$

*where*

$$\mathrm{SE}\ \left(\ln\ \left[\mathrm{IRR}\right]\right)=\surd \left(1/\left[\mathrm{incidence}\ \mathrm{rate}\ \mathrm{females}\right]+1/\left[\mathrm{incidence}\ \mathrm{rate}\ \mathrm{males}\right]-1/\mathrm{n}\ \left(\mathrm{females}\right)-1/\mathrm{n}\ \left(\mathrm{males}\right)\right)$$

Ethics approvals to conduct this study were granted by the Department of Defence and Veterans’ Affairs Human Research Ethics Committee (protocol 253–20) and the Human Research ethics Committees of Bond University and Charles Sturt University (protocols 253–20 & H20246, respectively).

## Results

A dataset of 12,385 work health and safety incident records was received the ADF data custodians, for incidents reported by Army personnel as having occurred in the two-year study period. From these, 338 were removed as they pertained to injuries affecting foreign military personnel, a member of the public, or a volunteer, and a further 363 were removed because the incident records did not indicate the personnel affected were Army personnel (the data in this regard were missing). A further 35 records were removed as they did not report meaningful incident data and 2899 were removed as they were categorised as a report of a near miss, exposure, dangerous incident, or fatality, rather than an injury. This left 8750 injuries, of which 8636 were classified as minor injuries (*n* = 1766 affecting female soldiers, *n* = 6870 male soldiers) and 114 as serious injuries (*n* = 19 affecting female soldiers, *n* = 95 male soldiers).

After accounting for the respective population sizes, the injury incidence rate for *minor* injuries was 20.75 minor injuries per 100 soldiers per year in females and 13.60 per 100 soldiers per year in males. Overall, female soldiers suffered minor injuries at a higher rate than male soldiers, with an associated IRR for minor injuries of 1.53 [95% CI = 1.46–1.60]. In similar fashion, the injury incidence rate for *serious* injuries was 0.22 serious injuries per 100 soldiers per year for female soldiers and 0.19 serious injuries per 100 soldiers per year for male soldiers, giving an IRR for serious injuries of 1.19 [95% CI = 0.73–1.94]. This indicates there was no significant difference between the sexes in incidence of *serious* injuries.

### Minor injuries

The distributions of injuries across commonly reported body locations differed only slightly between the sexes. The most common body location in which minor injuries occurred differed for female and male soldiers, with the ankle being the most common site in females and the knee in males (Table 1). In total, 14.4% of reported minor injuries were to the ankle in female soldiers, while 10.8% were to the knee. This is contrasted by 12.8% of minor injuries being at the knee in male soldiers, with a similar proportion of 12.5% of minor injuries affecting the ankle. The low back was the third most common site of minor injury for both males and females, accounting for 6.9 and 3.2% of reported minor injuries, respectively. The five most common anatomical locations of minor injury recorded for female soldiers in Table 1 comprised 43% of all reported minor injuries in female soldiers and 44% of all reported minor injuries in male soldiers. Female soldiers suffered a higher reported incidence rate of minor injury at all of the top 5 anatomical locations, except for the shoulder, where the minor injury incidence rates were not significantly different between the sexes.

**Table 1:** The most commonly reported body locations of minor injury in female, when compared to male australian soldiers

Location	Female	Male	IRR [95% CI]
Ankle	255 (3.00, 14.4%)	856 (1.69, 12.5%)	1.77 [1.54–2.03]
Knee	190 (2.23, 10.8%)	878 (1.74, 12.8%)	1.28 [1.10–1.50]
Low Back	122 (2.60 6.9%)	596 (1.18, 8.7%)	2.20 [1.81–2.67]
Foot	101 (1.19, 5.7%)	218 (0.43, 3.2%)	2.75 [2.17–3.48]
*Shoulder*	*101 (1.19, 5.7%)*	*535 (1.06, 7.8%)*	*1.12 [0.91–1.38]*
Collated others^a^	997 (11.71, 56.5%)	3787 (7.50, 55.1%)	1.56 [1.46–1.67]
**TOTAL**	**1766 (100%)**	**6870 (100%)**	**1.53 [1.46–1.60]**

^a^collated others < 90 injuries for females, < 213 injuries for males. Results reported as number of injuries (injuries/100 soldiers/year, % of injuries) IRR reference group = males

The same patterns for the most reported natures of minor injury were seen in both female and male soldiers, as evident in Table 2. Over 56% of reported minor injuries to female soldiers and 55% of reported injuries to male soldiers were soft tissue in nature. The second most common reported nature of minor injury in female soldiers was fractures, while lacerations were the second most common nature of minor injury in males. Females had a higher reported incidence rate for minor injury in all of the top five nature of injury categories, except for lacerations, for which the rate was not significantly different to that for males.

**Table 2:** The most commonly reported natures of minor injury in female, when compared to male australian soldiers

Nature	Female	Male	IRR [95% CI]
Soft tissue injury	994 (11.68, 56.3%)	3813 (7.55, 55.5%)	1.55 [1.44–1.65]
Fracture	116 (1.36, 6.6%)	331 (0.66, 4.8%)	2.08 [1.69–2.57]
Contusion, bruising and superficial crushing	97 (1.14, 5.5%)	270 (0.53, 3.9%)	2.13 [1.69–2.69]
Trauma to joints and ligaments	66 (0.78, 3.7%)	276 (0.55, 4.0%)	1.42 [1.09–1.85]
*Laceration or open wound*	*54 (0.63, 3.1%)*	*371 (0.73, 5.4%)*	*0.86 [0.65–1.15]*
Collated others^a^	439 (5.16, 24.9%)	1809 (3.58, 26.3%)	1.44 [1.30–1.59]
**TOTAL**	**1766 (100%)**	**6870 (100%)**	

^a^collated others < 50 injuries for females < 190 injuries for males. Results reported as number of injuries (injuries/100 soldiers/year, % of injuries) IRR reference group = males

The most common reported mechanisms of minor injuries in female soldiers are displayed in Table 3, along with rates at which they also occurred in male soldiers, for comparison. For both males and females, falls were the most common mechanism, followed by muscular stress without handling objects and muscular stress whilst handling objects. Females suffered a higher rate of minor injury than males, in all of the top 5 mechanism categories.

**Table 3:** The most commonly reported mechanisms of minor injury in female, when compared to male australian soldiers

Mechanism	Female	Male	IRR [95% CI]
Falls	446 (5.24, 25.3%)	1536 (3.04, 22.4%)	1.72 [1.56–1.91]
Muscular stress with no objects being handled	420 (4.93, 23.8%)	1422 (2.81, 20.7%)	1.75 [1.58–1.95]
Muscular stress while handling objects	283 (3.32, 16.0%)	1270 (2.51, 18.5%)	1.32 [1.17–1.50]
Contact with moving or stationary object	256 (3.01, 14.5%)	1124 (2.22, 16.4%)	1.35 [1.18–1.55]
Repetitive movement, low muscle loading	75 (0.88, 4.2%)	250 (0.49, 3.6%)	1.78 [1.38–2.30]
Collated others^a^	286 (3.36, 16.2%)	1268 (2.51,18.5%)	1.34 [1.18–1.52]
**TOTAL**	**1766 (100%)**	**6870 (100%)**	

^a^collated others < 32 injuries for females and < 160 injuries for males. Results reported as number of injuries (injuries/100 soldiers/year, % of injuries) IRR refence group = males

Physical training was the most common activity in which minor injuries occurred, in both female and male soldiers, accounting for almost 34.4 and 29.5% of reported injuries in female and male soldiers respectively (Table 4). This was followed by combat training and sport. The most common task being undertaken when minor injury occurred during physical training (PT) was similar for males and females, with running (♀ *n* = 111, 18.3% of PT minor injuries; ♂ *n* = 312,15.4%), followed by circuits (♀ *n* = 55, 9.1%; ♂ *n* = 186, 9.2%) being the two most common tasks associated with minor injury within PT. The third most common was the obstacle course for females (*n* = 41; 6.8%) and cardio training for males (*n* = 144; 7.1%). Within the category of sport, soccer was the most common sport type in which minor injuries were reported (*n* = 22; 17.9% of sport minor injuries), followed by volleyball (*n* = 16; 13.0%) and then netball (*n* = 15; 12.2%) in female personnel. In male personnel, soccer was also the most common sport type in which injuries occurred (*n* = 152; 21.9% of sport minor injuries) followed by touch football (*n* = 105; 15.1%) and then rugby union (*n* = 80; 11.5%). Sport was the only activity with similar minor injury rates for female and male soldiers.

**Table 4:** The most commonly reported activities causing minor injury in female, when compared to male australian soldiers

Activity	Female	Male	IRR [95% CI]
Physical Training	607 (7.13, 34.4%)	2025 (4.01, 29.5%)	1.78 [1.63–1.94]
Combat Training	259 (3.04, 14.7%)	1325 (2.62, 19.3%)	1.16 [1.02–1.32]
*Sport*	*123 (1.45, 7.0%)*	*694 (1.37, 10.1%)*	*1.05 [0.87–1.27]*
Walking	72 (0.85, 4.1%)	181 (0.36, 2.6%)	2.36 [1.80–3.10]
General Duties	55 (0.65, 3.1%)	152 (0.30, 2.2%)	2.15 [1.58–2.92]
Collated others^a^	650 (7.64, 36.8%)	2493 (4.93, 36.3%)	1.55 [1.42–1.68]
**TOTAL**	**1766 (100%)**	**6870 (100%)**	

^a^collated others < 50 injuries for females, < 90 injuries for males. Results reported as number of injuries (injuries/100 soldiers/year, % of all injuries) IRR reference group = males

### Serious injuries

The body locations in which SIs occurred most frequently in female soldiers are detailed in Table 5. The most common body location for SI in female personnel was the circulatory system, and this was much less common among serious injuries in males (Table 5). Two of the five circulatory system SIs in females were due to heat stress/stroke, with the other three due to exposure to biological factors or chemicals. In female soldiers, the forearm was the second most common body location of serious injuries, and both of the reported injuries at this body location were fractures. The proportion of serious injuries in males that affected the forearm was not significantly different to that in females. Other body sites were involved in no more than one serious injury each in female soldiers, and included the ankle, fingers, thumb, head, multiple systemic conditions, respiratory system, heart, pelvis and ‘not entered’. The body sites of two serious injuries in female soldiers were not specified. The second, third and fourth most common body locations for SI in males were specified as ‘systemic’ (*n* = 8, 8.4% of serious injuries), the shoulder (*n* = 7, 7.4%) and the knee (*n* = 6, 6.3%); none of these body sites accounted for more than 1 serious injury in female soldiers. The systemic serious injuries reported by male soldiers were typically poisoning, bites and stings, and serious injuries affecting the shoulder and knee were typically dislocations, fractures, or ligament tears.

**Table 5:** The most commonly reported locations of serious injury in female, when compared to male australian soldiers

Location	Female	Male	IRR [95% CI]
Circulatory System	5 (0.06, 26.3%)	4 (0.01, 4.2%)	7.42 [1.99–27.43]
Forearm	2 (0.02, 10.5%)	4 (0.01, 4.2%)	2.97 [0.54–16.20]
Collated others^a^	11 (0.13, 57.9%)	87 (0.17, 91.6%)	0.75 [0.40–1.40]
**TOTAL**	**19 (100%)**	**95 (100%)**	

^a^collated others n ≤ 1 injuries for females, n ≤ 5 injuries for males. Results reported as number of injuries (injuries/100 soldiers/year, % of all injuries) IRR reference group = males

Fractures were the most common nature of SI for both female and male soldiers (Table 6). The equal second most common natures of serious injuries among female soldiers were lacerations and heat stress/stroke. Other natures of injury accounted for no more than one serious injury each, in female soldiers. None of the specified natures of serious injury were experienced significantly more in one sex than the other.

**Table 6:** The most commonly reported natures of serious injury in female, when compared to male australian soldiers

Nature	Female	Male	IRR [95% CI]
Fracture	6 (0.07, 31.6%)	20 (0.04, 21.1%)	1.78 [0.72–4.43]
Laceration	2 (0.02, 10.5%)	8 (0.02, 8.4%)	1.48 [0.32–6.99]
Heat Stress/stroke	2 (0.02, 10.5%)	6 (0.01, 6.3%)	1.98 [0.40–9.80]
Collated others^a^	9 (0.11, 47.4%)	61 (0.12, 64.2%)	0.88 [0.44–1.76]
**TOTAL**	**19 (100%)**	**95 (100%)**	

^a^collated others n ≤ 1 injuries for females, n ≤ 2 injuries for males. Results reported as number of injuries (injuries/100 soldiers/year, % of all injuries) IRR reference group = males

The most common mechanism for SI in female soldiers was falls, which was also the equal second most common mechanism for serious injuries in male soldiers (.

Table 7). The most common in males was contact with objects (*n* = 17) which is not shown in Table 7. Being trapped by objects such as doors or windows was the second most common mechanism of SI in females, which was due to complicated fractures of fingers while closing windows or doors. Exposure to heat, as the third most common mechanism of SI in females, was also relatively common in males (Table 7).

**Table 7:** The most commonly reported mechanisms of serious injury in female, when compared to male australian soldiers

Mechanism	Female	Male	IRR [95% CI]
Falls	4 (0.05, 21.1%)	11 (0.02, 11.6%)	2.16 [0.69–6.78]
Being trapped by objects	3 (0.04, 15.8%)	5 (0.01, 5.3%)	3.56 [0.85–14.90]
Exposure to heat	2 (0.024, 10.5%)	8 (0.016, 8.4%)	1.48 [0.32–6.99]
Food Poisoning	2 (0.02, 10.5%)	0 (0.00)	
^a^Collated others	9 (0.11, 47.4%)	71 (0.14, 74.7%)	0.75 [0.38–1.50]
**TOTAL**	**19 (100%)**	**95 (100%)**	

﻿^a^﻿collated others n ≤ 1 injuries for females, < 11 injuries for males. Results reported as number of injuries (injuries/100 soldiers/year, % of all injuries) IRR reference group = males

The most commonly reported activity giving rise to SI in female soldiers was ‘eating’, with the two resulting injuries due to food poisoning (Table 8). The most common activity causing SI in males was physical training (15.8%), and this was followed by combat training (14.7%) in males. These and other activities accounted for no more than one SI each, in female soldiers.

**Table 8:** The most commonly reported activities causing serious injury in female, when compared to male australian soldiers

Activity	Female	Male	IRR [95% CI]
Eating	2 (0.02, 10.5%)	0	–
^a^Collated others	17 (0.20, 89.5%)	95 (0.19, 100%)	1.06 [0.63–1.78]
**TOTAL**	**19 (100%)**	**95 (100%)**	

^a^collated others n ≤ 1 injuries for females, < 4 injuries for males. Results reported as number of injuries (injuries/100 soldiers/year) IRR reference group = males

## Discussion

The aim of this study was to investigate differences in reported injury incidence rates and types between female and male soldiers in the Australian Army. Overall, it appears female soldiers report a 50% higher rate of minor injuries than male soldiers. However, rates of reported serious injuries do not appear to differ significantly between female and male soldiers. Small differences between the sexes in anatomical locations most commonly reported for injuries were found, which may assist to inform injury reduction strategies specifically for female soldiers.

Injury rates in general are typically higher in female military personnel than in male personnel both during training [16, 20] and on deployment [21, 22]. Some of this disparity may be explained by reporting, as female personnel are more likely to report an injury than males [23], and studies have shown that injury rates are similar between the sexes when reported and non-reported injuries were both taken into account [18]. The injury data capture system used by the ADF relies on self-report, and this may have influenced results of this study. Further evidence regarding the true differences or similarity in injury rates between the sexes may lie in the observed similar injury rates for serious injuries in female and male soldiers. Injuries which are more severe and require hospitalisation are highly likely to be reported and will be captured at point of care, as opposed to relying on self-report, which is known to underestimate injuries [24, 25]. The similar rates of serious injuries found in this study therefore suggest that true underlying injury rates (including rates of minor injuries) may not actually be dissimilar between the sexes, within Army.

Another explanation for the observed difference in minor injury rates between soldiers of each sex may be fitness. Studies which have adjusted analyses for fitness levels have found similar injury rates between female and male military personnel [11, 14, 15], and that females typically enter military training at a lower level of fitness than their male counterparts and relative to their own potential [15]. Evidence for this may lie in the greater improvements in fitness among female soldiers during basic training, when compared to male soldiers [15], and in the fact that injury rates tend to equalise at latter stages of a military career [16, 17].

Injuries to the lower limb, which are soft tissue in nature, suffered during both physical training and combat training are a common finding within military populations [6, 26–28]. Lower limb injury rates in army personnel in particular have been reported to be higher than in other services, thought to be due to greater exposure to risk while training in the field [29], such as risk associated with rugged, hilly and often rock covered terrain [30]. Of interest is the finding in this study that the ankle was the most commonly injured location in females, while the knee was the most commonly injured among males, though it should be noted rates of injuries at each of these body locations were also relatively high in the other sex. Ankle injuries are prevalent in military personnel, with estimations that they are suffered at a rate five times higher than that seen in the general population [31]. Rates of ankle injury for female cadets at the US military academy have been reported to be twice as high as those for male cadets [32] and female soldiers in the British Army were also found to suffer more foot and ankle sprains than males [33]. It has been proposed that this ankle sprain propensity in female military personnel may be due to a greater level of flexibility in females when compared to males [34]. Ill-fitting equipment, such as boots, may also play a role in this difference. Military equipment is often based on the male form, with female equipment simply being constructed as a smaller variant of the male equipment, without additional design consideration [35]. Boots for example, often do not have the required variants of foot width in smaller sizes, which may contribute to issues with the boot-foot interface in female personnel [10]. Additionally, initial ankle sprain was found to increase the risk of subsequent ankle sprains in a military population in the study by Kucera et al., [36], highlighting the necessity of prevention of initial injury where appropriate and ensuring adequate rehabilitation of injuries when they do occur.

The similarities between the sexes in rates of minor injuries in sport and injuries at the shoulder despite higher rates in female soldiers in other contexts may represent true similarities. Previous investigations in this army population have found a high number of knee and shoulder injuries in soccer, rugby league and rugby union, and a high number of ankle injuries in netball [7]. The traditional (though changing) common gravitation of female personnel to netball and male personnel to soccer and rugby may play a role in this finding. Likewise, the similar (rather than lower, as for other natures of injury) rate of lacerations in male soldiers, thought to be common due to traversing through vegetation or obstacle courses, may be due to a higher representation of males in combat units, which routinely perform these actions [37]. Roles in these positions may include unarmed combat, bayonet assault training and munitions handling, which are known to cause laceration injuries [38].

Many of the other locations, mechanisms, activities, and natures of injury were similar, in terms of their ranked position based on how commonly they occurred in soldiers of each sex. This may allow a degree of similarity and effectiveness of injury reduction strategies between sexes.

Despite rates of minor injuries being higher in female soldiers, rates of more serious injuries were similar between the sexes. Previous investigations have found a higher rate of serious injury amongst males when compared to females [39], especially while on deployment [2, 40]. Male military personnel have been found to suffer more knee ligament injuries in both cadets [41] and active duty personnel [42] than female personnel. This may be due to a greater level of participation in competitive sport within military service [42], more males being in infantry service of a higher physical nature, or greater exposure to direct combat while on deployment [2].

Of concern is the number of injury records in which specific data elements were not entered, especially for serious injuries. Serious injuries may come at a risk of medical discharge, permanent disability, and other long term health ramifications, highlighting the necessity of recognizing them in the first instance, prior to initiating strategies to reduce them [9]. Efforts to reduce these injuries are ineffective if they are ill informed, and missing data detracts from the available information from which to determine preventive strategies.

This study is limited by the self-reporting nature of the injury capture system utilised in this population. This method may be prone to recall bias, and some injuries may go completely unreported. Likewise, females are known to report injuries more readily and sooner than males, which may have affected the comparative minor injury rates, particularly. It was not possible to capture confounding variables such as fitness, which is a known risk factor for injury. Studies of this nature which adjust for fitness have found minimal differences in injury rates between female and male soldiers. The study period also included data from a timeframe of the COVID-19 response, whereby priorities and tasks may have been changed, including training practices and deployments to border checkpoints. Another limitation of the study is the relatively small number of SIs suffered by female soldiers. This may have affected the precision of the estimates of injury rates, evidenced by the large confidence intervals in some calculations.

## Conclusion

Female soldiers of the Australian Army reported higher incidence rates for minor injuries than their male counterparts but similar rates for serious injuries. Injuries to the ankle were the most common in females, while the knee was the most common in males. Due to the subtle differences in the profiles of minor and serious injuries between female and male soldiers, injury reduction strategies may need to be varied to ensure effectiveness for both sexes.

## Data Availability

The data that support the findings of this study are available from the Department of Defence, but restrictions apply to the availability of these data, which were used under license for the current study, and so are not publicly available. Data are however available from the authors upon reasonable request and with permission of the Department of Defence.

## Abbreviations

ADF: Australian Defence Force
ARA: Australian Regular Army
CI: Confidence Interval
IR: Incidence Rate
IRR: Incidence Rate Ratio
MI: Minor Injury
PT: Physical Training
SI: Serious Injury
